# Rapid sampling of local minima in protein energy surface and effective reduction through a multi-objective filter

**DOI:** 10.1186/1477-5956-11-S1-S12

**Published:** 2013-11-07

**Authors:** Brian S Olson, Amarda Shehu

**Affiliations:** 1 Department of Computer Science, George Mason University, 4400 University Dr., Fairfax, VA, 22030, USA; 2 Department of Bioengineering, George Mason University, 4400 University Dr., Fairfax, VA, 22030, USA; 3 School of Systems Biology, George Mason University, 10900 University Blvd., Manassas, VA, 20110, USA

## Abstract

**Background:**

Many problems in protein modeling require obtaining a discrete representation of the protein conformational space as an ensemble of conformations. In ab-initio structure prediction, in particular, where the goal is to predict the native structure of a protein chain given its amino-acid sequence, the ensemble needs to satisfy energetic constraints. Given the thermodynamic hypothesis, an effective ensemble contains low-energy conformations which are similar to the native structure. The high-dimensionality of the conformational space and the ruggedness of the underlying energy surface currently make it very difficult to obtain such an ensemble. Recent studies have proposed that Basin Hopping is a promising probabilistic search framework to obtain a discrete representation of the protein energy surface in terms of local minima. Basin Hopping performs a series of structural perturbations followed by energy minimizations with the goal of hopping between nearby energy minima. This approach has been shown to be effective in obtaining conformations near the native structure for small systems. Recent work by us has extended this framework to larger systems through employment of the molecular fragment replacement technique, resulting in rapid sampling of large ensembles.

**Methods:**

This paper investigates the algorithmic components in Basin Hopping to both understand and control their effect on the sampling of near-native minima. Realizing that such an ensemble is reduced before further refinement in full ab-initio protocols, we take an additional step and analyze the quality of the ensemble retained by ensemble reduction techniques. We propose a novel multi-objective technique based on the Pareto front to filter the ensemble of sampled local minima.

**Results and conclusions:**

We show that controlling the magnitude of the perturbation allows directly controlling the distance between consecutively-sampled local minima and, in turn, steering the exploration towards conformations near the native structure. For the minimization step, we show that the addition of Metropolis Monte Carlo-based minimization is no more effective than a simple greedy search. Finally, we show that the size of the ensemble of sampled local minima can be effectively and efficiently reduced by a multi-objective filter to obtain a simpler representation of the probed energy surface.

## Background

Many problems in protein modeling demand obtaining a discrete representation of the protein conformational space in terms of an ensemble of conformations. In the ab-initio structure prediction problem, in particular, where the goal is to predict the native structure of a protein chain given its amino-acid sequence, the ensemble needs to satisfy certain energetic constraints. Under the thermodynamics treatment [1], the native structure is located at the basin of a funnel-like energy surface [2,3]. Thus, search algorithms that generate conformations and are guided towards low-energy ones by a potential energy function should obtain an effective ensemble containing low-energy conformations near the native structure. This is predominantly not the case due to the size and high-dimensionality of the protein conformational space and the ruggedness of the underlying energy surface [4]. Despite these challenges, the rapidly growing gap between the wealth of protein sequence data and the relatively sparse set of experimentally-determined native protein structures necessitates research into computational approaches to determining protein structure. The ability to deter-mine structural information through ab-initio computational methods promises to elucidate the relationship between protein structure and function and advance studies of biological function and drug design. [5-7].

The two predominant reasons that it is challenging to obtain a conformational ensemble near the (unknown) native structure of a protein are poor sampling capability by the search algorithm and inaccuracies in the energy function employed by this algorithm to probe low-energy regions of the energy surface. Limited sampling capability is to be expected when considering a vast high-dimensional search space. For the purpose of illustrating this point, consider a protein chain of *n *amino acids. Each amino acid contains a group of atoms. A shared subset among all known amino acids, known as backbone atoms, defines the main backbone thread that runs through the protein chain. Even if focusing on modeling only this thread and its spatial arrangements, which we refer to as conformations, the space populated by these conformations has many dimensions. There are 4 heavy backbone atoms per amino acid. A cartesian representation would define a 4 * 3*n*-dimensional space. One can reduce this down to a 3*n*- or a 2*n*-dimensional space if instead of maintaining cartesian coordinates, only backbone dihedral angles are maintained to represent a conformation. For a small protein of 30 amino acids, the conformational space has at least 60 dimensions in this angular representation.

The high-dimensionality of the search space favors certain approaches to the problem of obtaining an ensemble of conformations near the native structure in a reasonable amount of time. Methods based on the Molecular Dynamics (MD) approach simulate the actual folding process where a protein slowly tumbles down the energy surface from its unfolded to the folded native state. Simulating folding kinetics demands very small moves in the energy surface in order to retain accuracy when integrating equations of motions. For this reason, MD-based approaches demand significant computational resources (e.g., Folding@Home) and/or specialized hardware (e.g. Antoine) [8,9]. Conducting, instead, a global energy optimization which forgoes information of folding kinetics is useful and justified under the thermodynamics treatment. Approaches based on optimizing the energy of a conformation can obtain native conformations orders of magnitude faster than approaches that simulate folding pathways [10]. Many of these approaches follow the Monte Carlo (MC) approach in order to enhance their sampling capability over the MD approach. MC-based approaches, however, still struggle to obtain native conformations on medium-size proteins due to the complexity of the protein energy surface [4]. Due to this significant challenge, stochastic optimization algorithms for protein conformational search remains a very active field of research. [11].

Many stochastic optimization techniques for ab-initio structure prediction have converged on a unifying strategy of sampling of a large number of low-energy conformations. The emphasis on the size is due to the fact that many local minima may be present in the energy surface, particularly in those constructed by current functions available to measure the potential energy of a protein conformation. Sampled conformations are the end points of independent MD or MC trajectories which perform a local optimization on a given coarse-grained energy function. In full ab-initio protocols, stochastic optimization with a coarse-grained energy function constitutes only stage one. After the ensemble of low-energy conformations is obtained, often referred to as decoys, the decoy ensemble is reduced in preparation for a second stage of optimization. The reduction employs either filtering by energies or grouping by structural similarity through clustering-based techniques. The purpose of the reduction is to reveal a subset of conformations representing local minima that are worth optimizing further at greater structural detail and through some finer-grained energy function in order to improve their proximity to the native structure [5,12-17].

Optimization-based approaches are effective at obtaining native conformations for many small to medium size proteins, however, the accuracy of these approaches is ultimately bound by the accuracy of the employed energy function. State-of-the-art energy functions employ approximations to improve performance, but these approximations can lead to errors in the energy function which are responsible for deviations between the global minimum of the energy function for a particular protein and the experimentally-determined native structure for the protein [10,18]. Because of these deviations, approaches which sample a broad range of low-energy minima, rather than focussing on a single global minimum, are more appropriate for coarse-grained energy functions. These sampled minima can then be further scrutinized though additional heavy-duty optimization techniques.

In most MC-based methods, the broad view is obtained by launching many independent MC trajectories. In other approaches, the trajectories are integrated into a tree-based or population-based search framework, maintaining a broader view and thus a more diverse decoy ensemble by employing analysis of the ensemble to effectively guide the search towards relevant regions of the search space [19-22]. In robotics-inspired approaches, a tree of conformations grows in conformational space [19,20], and low-dimensional embeddings of the energy surface and conformational space are used to collect online statistics with which to adaptively bias the search towards low-energy regions and away from over-sampled regions. In evolutionary-inspired approaches [21,22], multi-objective analysis of energy terms is used to guide the search towards a diverse population of conformations. Currently, this multi-objective analysis is applied only to all-atom representations and applied to very small proteins.

None of the above methods explicitly sample local minima in the energy surface. Rather, they rely on some post-analysis to group conformations together to identify captured local minima. Recent studies by us and others have proposed that Basin Hopping (BH) is a promising stochastic optimization framework to directly obtain a discrete representation of the protein energy surface in terms of local minima [23-25]. The framework was originally introduced to obtain the Lennard-Jones minima of small atomic clusters [26]. The inspiration for the BH framework in [26] comes from evolutionary search algorithms, such as Iterated Local Search (ILS). ILS consists of iterated applications of perturbation followed by local search and is popular for solving discrete optimization problems [27]. An adaptation of ILS for molecular modeling introduces a Metropolis-like criterion to bias the sampling of local minima towards lower-energy regions of the search space.

Pervious realizations of the BH framework, most notably in the MC with Minimization algorithm, essentially differ in their implementation of the perturbation and minimization components [28,29]. The perturbation component typically directly modifies the atomic coordinates of a conformation and minimization is performed through a gradient descent or low-temperature Metropolis MC trajectory. Successful applications of BH algorithms include obtaining local minima of small atomic clusters, mapping the energy surface of polyalanines, and modeling of other small proteins [10,30-32].

In recent years, new attention has been given to BH as a framework for ab-initio protein structure prediction [23-25,33]. In [23], a particular realization of the BH framework on small proteins is shown to obtain both lower-energy minima and conformation closer to the experimentally-determined native structure than the MD with Simulated Annealing approach. Here conformations are perturbed by modifying atomic coordinates by small random values. Minimization is then implemented as a gradient descent over a coarse-grained energy function. While effective on small proteins, the performance of this implementation decreases significantly on sequences with more than 75 amino acids [23].

In recent work, we extend the effectiveness of BH to longer protein sequences by employing molecular fragment replacement with a coarse-grained energy function [24,25] (detailed in the Methods section). Experiments show that the resulting BH algorithm is able to sample conformations near experimentally-determined native structures as well as other state-of-the-art structure prediction protocols. This proposed coarse-grained sampling algorithm is intended to generate decoy conformations as the first step in a structure prediction protocol which then further refines selected decoy conformations.

Given the recent attention and promise of BH as a framework for protein structure prediction, a greater understanding of the effectiveness and efficiency of the key BH components is critical. While some studies into the efficacy of different perturbation moves for identifying low-energy isomers of small Si and CU clusters exist in the computational physics community [34], no such study is available for proteins.

In this work we offer a detailed analysis of the BH framework in the context of structure prediction. We provide an in-depth analysis of BH's two key components, perturbation and minimization, and show how adjusting these components affects sampling of decoy conformations. Controlling the magnitude of each perturbation allows us to directly control the distance between consecutively-sampled local minima. We show that this local-minima distance is directly related to the ability of the BH algorithm to effectively explore the conformational space and obtain conformations near the native protein structure. We also explore the use of temperature when employing Metropolis MC minimization and show that a shorter greedy search is just as effective as a more intensive Metropolis MC minimization.

Our BH algorithm is effective at rapidly sampling large numbers of decoy conformations that represent local minima in the protein energy surface. Here we extend analysis of this decoy ensemble beyond simply comparing the decoys with the lowest lRMSD to the experimentally-determined native structure. Realizing that the true utility of a stochastic optimization technique is in which subset of its conformations would be retained for further refinement in a complete ab-initio protocol, we pursue different reduction techniques and analyze how each of those would retain near-native conformations sampled by the BH algorithm.

We show, as expected, that ensemble reduction techniques based on total energy miss many promising near-native conformations. This is to be expected, as a method with high sampling capability will uncover many low-energy non-native conformations. Given the growing knowledge that current energy functions, particularly coarse-grained ones, are weakly funneled, displaying very weak correlation between low energies and proximity to the native structure, no energetic threshold will discard non-native and retain near-native conformations. Our analysis shows this on 15 diverse protein systems. On the other hand, reduction techniques that discard energies and instead cluster conformations by structural similarity can be quite computationally demanding with large ensemble sizes (106 conformations or more). Such techniques would also not be viable if there is a need to possibly apply them repeatedly during search.

We introduce here a novel energy-based ensemble reduction technique that makes use of multi-objective analysis to enhance retention near-native decoys. The technique decomposes the energy of each conformation into the various terms in the energy function and evaluates conformations based on Pareto count and the Pareto front. The analysis is particularly suited to finding a subset of conformations that satisfy conflicting terms, as is the case with terms added up in energy functions. We show that our Pareto-based selection scheme significantly reduces the size of the decoy ensemble, while retaining a more diverse set of near-native conformations than employing a total energy threshold. These results are shown to be robust and valid when using two different state-of-the-art coarse-grained energy functions commonly employed in a structure prediction setting. The computational complexity of computing these multi-objective metrics makes them practical, even on very large ensembles of decoy conformations. Since the Pareto front and Pareto count can be computed online, these multi-objective energy metrics are also ideal to be employed in online analyses used by tree-based and population-based search algorithms to adaptively guide search.

A preliminary investigation of this ensemble reduction technique was presented in [33]. In this work, we extend the BH framework to employ two different state-of-the-art energy functions and analyze the effectiveness the of ensemble reduction technique on the energy surface sampled by both energy functions.

## Methods

Obtaining a broad view of the energy surface for a protein sequence of interest in the coarse-grained stage relies on a stochastic optimization algorithm to go through different conformations and an energy function to score these conformations and guide the search towards low-energy ones. As described in the Background section, coarse graining in this stage refers to the employment of a coarse-grained representation for the protein chain. As in many state-of-the-art ab-initio protocols, we employ an extended backbone representation in our BH-based algorithm, sacrificing side chains. This representation is detailed first below, in the Molecular representation section. Given a coarse-grained representation, a coarse-grained energy function scores conformations generated by the search algorithm. We consider here two state-of-the-art coarse-grained energy functions, the AMW and the Rosetta energy functions, briefly described below in the Coarse-grained energy function section. The BH-based stochastic optimization algorithm that makes use of the chosen representation and energy function(s) is described next, followed by details on the different implementations considered and analyzed for its perturbation and minimization components. The implementations for the algorithmic components of the algorithm are analyzed in detail for how they affects the quality of the (decoy) ensemble of local minima produced by the algorithm. The Pareto-optimal filtering of this ensemble is described last.

### Molecular representation

The structural detail in the side chains of a protein is largely sacrificed in the interest of expediency. It is worth noting that once the decoy ensemble is obtained and reduced through selection techniques, the retained coarse-grained conformations are added structural detail through side-chain packing techniques [35,36]. The AMW and the Rosetta coarse-grained energy functions considered here and described below operate on slightly different extended backbone representations. In both cases, the backbone heavy atoms *N *, *C*, *Cα*, and *O *are explicitly modeled. When using AMW, side-chains are reduced to only the *Cβ *atom (with exception of glycine, where there is no such atom). When using Rosetta, a side chain is reduced to a pseudo-atom centered at the side chain's centroid.

Cartesian coordinates for the atoms modeled are employed by the respective energy functions to associate a potential energy value or score with a generated conformation. Internally, the representation employed by the algorithm to generate conformations maintains only three backbone dihedral angles (*ϕ*, *ψ*, *ω*) per amino acid. This angular representation, also known as a kinematic model, is based on the idealized geometry assumption, which fixes bond lengths and angles to idealized (native) values (taken from CHARMM22 [37]) and limits variations to backbone dihedral angles. Using this angular representation, the BH algorithm essentially generates conformations by replacing values for an entire block of *ϕ*, *ψ*, *ω *angles of *f *consecutive amino acids at a time (*f *is often referred to as the fragment length). New values for a block are sampled from a fragment configuration library, which essentially stores blocks of angles observed in known native structures, as described in the Background section. After a conformation is obtained in its angular representation, forward kinematics is employed to obtain cartesian coordinates for the modeled atoms from the backbone dihedral angles [38].

### Coarse-grained energy function

Our experiments in this paper consider two state-of-the-art coarse-grained energy functions, the Associative Memory Hamiltonian with Water (AMW), and the Rosetta energy function, described below.

#### AMW energy function

This coarse-grained potential, originally proposed in [39], has been used by us and others in the context of different search procedures for the purpose of decoy sampling in ab-initio structure prediction [12,19,20,40-42]. Briefly, AMW sums 5 non-local terms (local interactions are kept at ideal values under the idealized geometry assumption): *E*_AMW _= *E*_Lennard*-*Jones _+ *E*_H*-*Bond _+ *E*_compaction _+ *E*_burial _+ *E*_water_. The *E*_Lennard*-*Jones _term is implemented after the 12-6 Lennard-Jones potential in AMBER9 [43] allowing a soft penetration of van der Waals spheres. The *E*_H*-*Bond _term allows modeling hydrogen bonds and is implemented as in [44]. The other terms, *E*_compaction_, *E*_burial_, and *E*_water_, allow formation of a hydrophobic core and water-mediated interactions (See [12] for more details).

#### Rosetta energy function

The Rosetta energy function we use here corresponds to the *score3 *setting in the suite of energy functions used in the Rosetta ab-initio protocol [45]. The different energy functions used in the Rosetta ab-initio protocol are scaled versions of a full energy function that is a linear combination of 10 terms. These terms measure repulsion, amino-acid propensities, residue environment, residue pair interactions, interactions between secondary structure elements, density, and compactness. The different substages used in the Rosetta ab-initio protocol use subsets of the terms of the full energy function and modify weights in the linear combination to promote certain interactions over others. We use here the score3 setting, as this corresponds to the full coarse-grained Rosetta energy function.

### Probabilistic Search Algorithm based on Basin Hopping Framework

We first proposed the BH-based probabilistic search algorithm that we analyze in detail in this paper in [25]. The algorithm iteratively hops between consecutive minima *C_i _*and *C_i_*_+1 _by performing a perturbation followed by a minimization. Conformation *C_i _*is perturbed to obtain a new higher-energy conformation *C*_perturb,i _which allows the search to escape from its current local minimum. *C*_perturb,i _is then minimized through a series of small modifications until a new minimum *C_i_*_+1 _is reached. The Metropolis criterion is then employed to determine whether or not the current state of the trajectory is moved to *C_i_*_+1 _based on the energetic difference between *C_i _*and *C_i_*_+1_. This results in a trajectory of conformations representing local minima in the energy surface. The Metropolis criterion guides the trajectory towards lower-energy regions of the energy surface. Thus, the ensemble of decoy conformations obtained with BH consists of good-quality conformations that represent local minima in the protein energy surface.

The two main components in the algorithm are the perturbation and minimization. They both modify conformations using the molecular fragment replacement technique described in the Background section. Briefly, given a conformation, a trimer (three consecutive amino acids) is selected at random over the target protein sequence. A configuration for that trimer (consisting of 9 backbone dihedral angles - *ϕ*, *ψ*, *ω *for each of the amino acids in the trimer) is then obtained at random over the available ones in a fragment configuration library. The library is pre-compiled from configurations extracted from known non-redundant native structures. The fragment configuration library is constructed as in the protocol outlined in the Rosetta ab-initio package (for further details, cf. to Ref [25]). While the perturbation replaces one trimer configuration, the minimization consists of repeated replacements until a certain preset number of consecutive attempts fail to lower energy.

In this work we propose and analyze different implementations for the minimization and perturbation components, paying attention to how they affects the quality of the decoy ensemble. We do not explicitly analyze the efficacy of different moves that one can employ in perturbation. Comparative results between work in [23], which applies small random perturbations to atomic coordinates, and work in [25], which applies trimer configuration replacements, suggests that the latter moves are more efficient with growing sequence length and confer higher sampling capability.

### Perturbation

In order to effectively explore the conformational space, the magnitude of the perturbation must be large enough to escape the current local minimum, but not so large that consecutively sampled local minima are too unrelated in the conformational space. If the perturbation magnitude is too small, the minimization step is likely to return to the previous local minima. Even if a new minima is reached, if the average distance between C_i _and C_i+1 _is too small, then the search will be too inefficient to cover the breadth of the protein conformational space. If the perturbation magnitude is too larger, however, then the search effectively samples local minima at random over the entire energy surface and cannot be effectively guided by the Metropolis criterion towards lower-energy regions.

Perturbation is performed through a single trimer fragment replacement on *C_i _*to obtain *C*_perturb,i_. Since the magnitude of each perturbation (measured as the lRMSD between *C_i _*and *C*_perturb,i_)) varies based on which fragment configuration is selected from the fragment library, the following technique is employed to explicitly bias the magnitude of each perturbation to a configured value *D*. For each perturbation, a target magnitude *d *is sampled from a gaussian distribution centered at *D *with a standard deviation of 1. New perturbed conformations are then sampled through fragment replacement until a conformation *C*_perturb,i _is found which is *d *Å lRMSD from *C_i _*(within a tolerance *t*). If *n *attempts have been made without finding a conformation which satisfies the target perturbation magnitude, then the conformation which comes closest to satisfying this target is used as *C*_perturb,i_. The value of *n *is set to 20, which is large enough that a conformation *C*_perturb,i _can be found within a tolerance of *t *= 0.5Å in nearly every case. Since only the final conformation selected for *C*_perturb,i _is evaluated for energy, this process of sampling multiple perturbation candidates does not add significant computational time to the overall algorithm.

### Minimization

The minimization component maps a perturbed conformation to a nearby local minima in the protein energy surface through a series of small modifications. Since the minimization step consumes the vast majority of the computational resources in a BH algorithm, it is important to balance the efficiency of a minimization technique with its effectiveness at probing local minima. In this work we compare the more computationally efficient greedy search, summarized above and implemented originally in [25], to a Metropolis MC (MMC) search for minimization. While MMC is more computationally intensive, it is able to probe deeper into local minima by adjusting the effective temperature of the search. We do not investigate gradient-based techniques, as they converge very slowly to a local minimum [23].

In a greedy search only modifications (referred to here as moves) are made which lower the energy of the conformation. An MMC search, however, will occasionally accept a move which raises the energy of the conformation in order to cross over an energetic barrier. The height of the energetic barrier which can be crossed is controlled by the effective temperature, *T *, employed by the metropolis criterion. By setting *T *to a small non-zero value, the MMC search can effectively jump over low energy barriers while remaining the in same local energy funnel. This allows a MMC search to reach deeper local minima than a greedy search which can get stuck on on these low energetic barriers.

Probing down to true local minima in the protein energy surface can be computationally intensive and analysis of the AMW energy surface in previous work shows that experimentally-determined native structures are found somewhere above their corresponding true minima [25]. For this reason, each MMC minimization is run until only *k *consecutive moves are rejected. For the purposes of this study, the working definition of a local minima is thus determined by the value of *k*. Based on previous work, *k *is set to the length of the target protein sequence which is sufficient to sampled near-native conformations [25].

The temperature parameter, *T *, effectively controls the height of energy barriers which can be crossed by the MMC minimization. A higher value of *T *makes it less likely that the minimization will get stuck, and thus, on average, more MMC moves will be made before reaching the termination condition of *k *consecutive failed moves. In the special case where *T *= 0, the MMC search is effectively equivalent to the greedy search shown effective in our previous work [25]. In the Results section, we compare the effectiveness of greedy vs. MMC search in minimization. Three different effective temperatures are studied in the context of the MMC search. Temperatures, *T*_0_, *T*_1_, and *T*_2_, correspond to a 0.1 probability of accepting energy increases of 1.4, 1.7, and 2.6 kcal/mol, respectively.

### Multi-objective ensemble reduction

The ensemble Ω of local minima that is obtained by the BH-based algorithm under some chosen implementations of the perturbation and minimization components can be large. In a complete ab-initio structure prediction protocol, a few promising coarse-grained structures are selected for refinement in greater structural detail. Therefore, the ensemble Ω produced by the BH framework must be reduced to a relevant subset of local minima conformations. Here a trade-off must be made between selecting a small number of conformations and selecting a diverse enough subset so as to increase the likelihood of retaining near-native conformations.

A simple ensemble reduction technique which retains all conformations with an energy below a given threshold is problematic because there is no accepted method for selecting an appropriate threshold for an arbitrary protein system. Using the threshold method, it is likely that the reduced ensemble will either be too large to make fine-grained refinement practical or that many near-native conformations will be excluded due to noise in the energy function (recall that current energy functions are all weakly funneled and thus global minimum may not correspond to the native structure). By comparing energy terms individually, however, a more nuanced energetic comparison can help remove some of the noise inherent in energy functions which results from the weighted linear combination of unrelated energy terms [46]. This multi-objective analysis is the foundation of the technique we propose and analyze here to reduce Ω.

**Figure 1:**
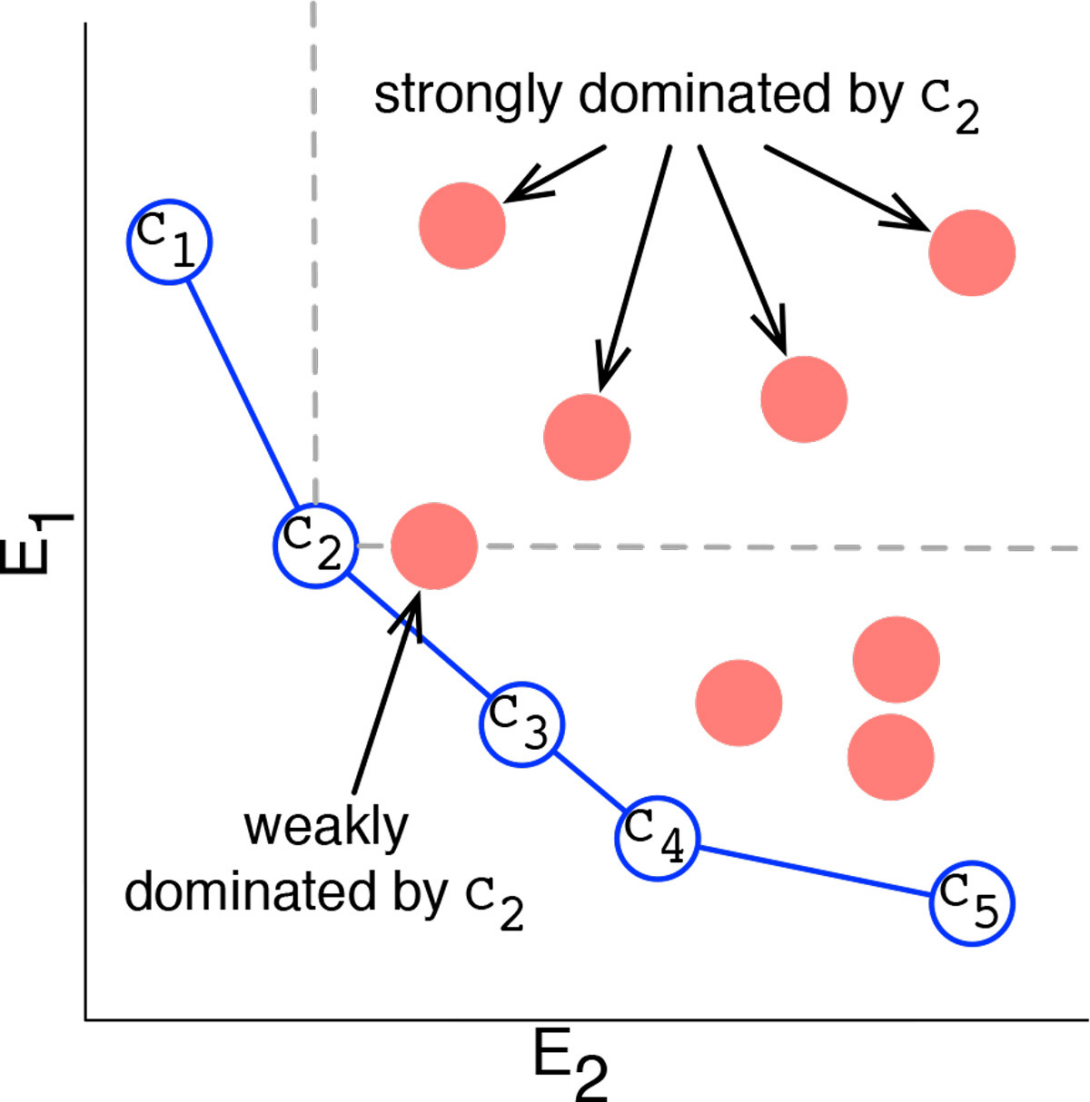
**Conformations are plotted with respect to two energy terms *E*_1 _and *E*_2_**. Conformations represented by empty blue circles are non-dominated and form the Pareto front. *C*_2 _strongly dominates 4 conformations and weakly dominates 1 additional conformation, thus the Pareto count of *C*_2 _is 4 for strong Pareto dominance and 5 for weak Pareto dominance.

A conformation *C_i _*is said to dominate a conformation *C_j _*when every energy term in *C_i _*is lower than the corresponding term in *C_j _*. *C_j _*is said to be non-dominated if there is no conformation in Ω that dominates *C_j _*. Conformations in the non-dominated ensemble, referred to as the Pareto front, are considered equivalent with respect to a multi-objective analysis. Figure 1 illustrates the the Pareto front for a simplified energy function containing only two terms.

When every term in *C_i _*is less than every term in *C_j _*, *C_i _*is said to strongly dominate *C_j _*. If the requirement for dominance is relaxed such that every term in *C_i _*is less than or equal to its corresponding term in *C_j _*, this is referred to as weak dominance. Typically, multi-objective analysis employs strong dominance, however, in some cases weak dominance may be more appropriate, particularly if one of the energy terms has a very low variance.

Membership in the Pareto front is a binary state. It is often desirable to employ multi-objective analysis to rank conformations whether or not they lie in the Pareto front. One such metric is the Pareto count of a conformation. The Pareto count of *C_i _*measures the number of other conformations *C_i _*dominates. Pareto count is illustrated in Figure 1.

This work employs employs multi-objective analysis as a method for filtering the Ω ensemble of conformations representing local minima. The ensemble Ω*_PF _*corresponds to conformations that lie in the Pareto front and Ω*_PC_*_(*n*) _corresponds to conformations with a Pareto count above a given threshold value. The variable *n *is set to a particular percentage of Ω and a Pareto count threshold is chosen such that *| *Ω*_PC_*_(*n*)_*| *= n * *| *Ω *|*. For example, Ω*_PC_*_(5%) _represents the 5% of conformations in Ω with the highest values for Pareto count.

## Results and discussion

**Experimental setup **The analysis is conducted over 15 target protein systems listed in Table 1 which range from 61-123 amino acids in length and cover the *α*, *β*, and *α/β *folds. Experiments are run for a fixed budget of 10,000,000 energy function evaluations. Since over 90% of CPU time is spent on such evaluations, the limit ensures a fair comparison between different parameter selections on a diverse set of proteins. Computing 10,000,000 energy function evaluations takes 1-4 days of CPU time on a 2.4Ghz Core i7 processor, depending on protein length. The perturbation and minimization components are analyzed first in the Analysis of BH framework section with respect to the AMW energy function. Lastly, the Multi-objective ensemble reduction section presents results for Ω ensembles obtained by running the BH framework with both the AMW and Rosetta energy functions.

**Table 1:** local search.

	Native PDB id	Size	fold	% *α*	% *β*	Lowest Energy (kcal/mol)				Lowest lRMSD (Å)			
						*T *= 0	*T*_0_	*T*_1_	*T*_2_	*T *= 0	*T*_0_	*T*_1_	*T*_2_
1	1dtdB	61	*α/β*	15	46	-128.2	-132.1	-131.6	-127.9	6.9	6.6	6.9	7.0
2	1isuA	62	*α/β*	15	19	-127.8	-130.3	-130.7	-130.2	6.3	6.0	6.4	6.0
3	1c8cA	64	*α/β*	22	48	-133.5	-134.8	-130.8	-129.6	6.5	6.6	7.4	7.3
4	1sap	66	*α/β*	30	44	-132.8	-132.3	-133.6	-127.3	6.5	6.0	6.8	6.9
5	1hz6A	67	*α/β*	31	42	-143.5	-144.7	-142.1	-138.9	5.7	5.9	6.0	6.0
6	1wapA	68	*β*	0	62	-118.4	-127.2	-133.9	-127.9	7.4	7.6	7.4	7.5
7	1fwp	69	*α/β*	30	26	-152.8	-152.0	-143.5	-143.2	6.3	6.7	6.5	6.1
8	1ail	70	*α*	84	0	-170.6	-171.0	-167.3	-168.4	3.2	3.2	3.4	3.3
9	1aoy	78	*α/β*	41	10	-183.9	-181.2	-180.8	-184.1	5.7	6.4	6.0	6.4
10	1cc5	83	*α*	47	4	-170.9	-171.5	-179.1	-173.8	5.8	5.7	5.8	5.8
11	2ezk	93	*α*	68	0	-217.3	-218.6	-224.4	-216.0	4.3	4.6	4.2	4.4
12	1hhp	99	*β*	7	48	-168.7	-175.4	-179.0	-175.9	10.4	10.4	10.0	10.5
13	2hg6	106	*α/β*	34	21	-233.6	-236.8	-239.5	-235.1	8.8	9.0	8.8	9.2
14	3gwl	106	*α*	70	0	-264.6	-270.4	-273.9	-267.3	4.9	4.9	4.4	5.2
15	2h5nD	123	*α*	71	2	-307.8	-313.0	-316.5	-313.2	7.5	7.9	7.4	8.1

Columns 2-4 show the native PDB id, size and fold topology for each of the 15 target protein systems. Columns 5 and 6 break the fold topology down as the percentage of amino acids which are part of *↵*-helices and *(3*-sheets. Columns 7-10 report the minimum energy achieved for each temperature *T *of the minimization component of the BH framework. Columns 11-14 then report the corresponding lowest lRMSD to the native structure achieved for each *T *.

### Analysis of BH framework

Analysis is performed on the effect of biasing perturbation distance and varying the temperature of the local search in the BH framework.

#### Biasing perturbation distance

**Figure 2:**
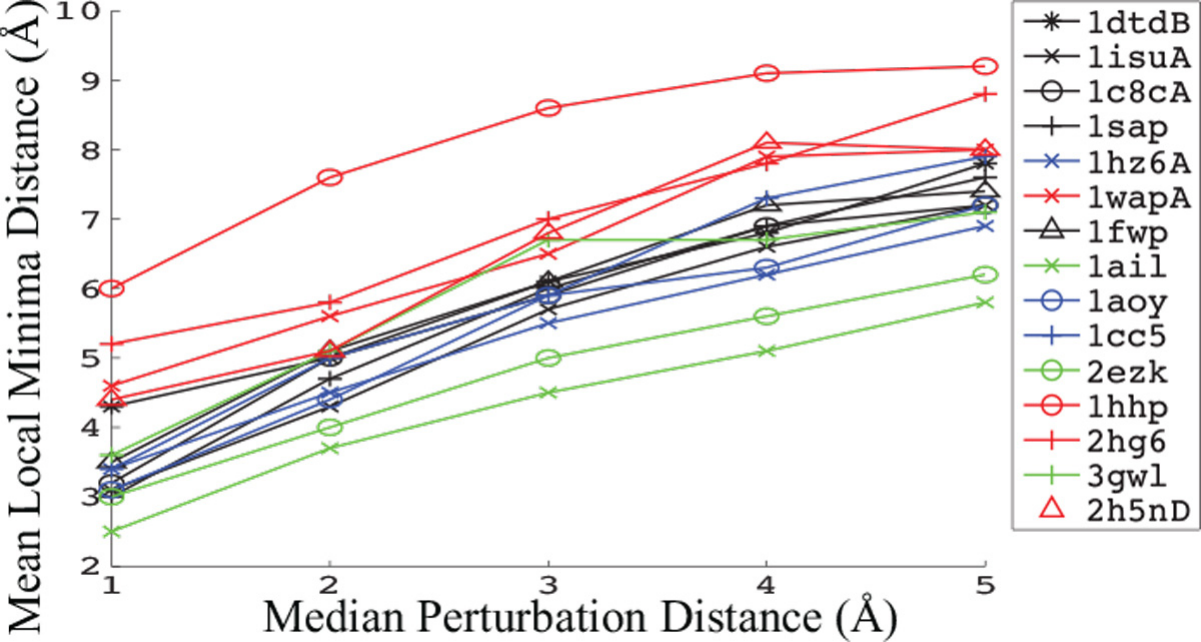
**The mean *μ_|MM | _*is shown for a given target perturbation distance *D*, where *μ|MM | *refers to the distance between two consecutively sampled local minima**.

**Figure 3:**
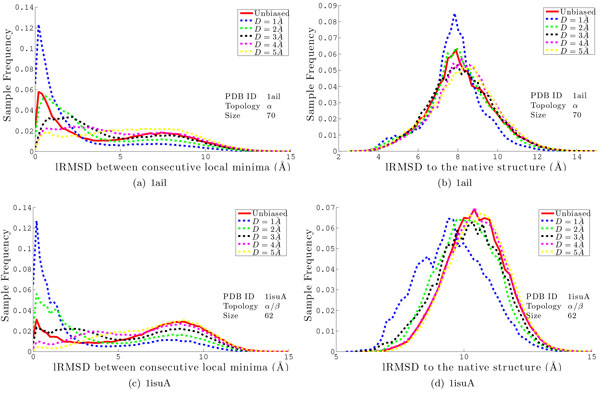
**The frequencies of *μ_|MM | _*sampled during the search for proteins with native structure PDB ids 1ail and 1isuA are shown in (a) and (c), respectively**. Frequency of lRMSDs to the native structure for each protein are given in (b) and (d), respectively. The solid red line represents BH employing the unbiased perturbation method. The dashed lines represent BH with median perturbation distances *D *= 1Å to *D *= 5Å.

Our previous work shows a direct correlation between the mean lRMSD between consecutive local minima (referred to from now on as *μ_|MM |_*) and the ability of the BH framework to sample near-native conformations [25]. Figure 2 shows that *μ_|MM | _*can be effectively controlled by biasing the magnitude of the perturbation jump through a target perturbation distance *D*; as *D *is increased, there is a corresponding increase in *μ_|MM |_*. Tuning *D *does not significantly effect the single lowest lRMSD conformation sampled (lRMSD measures the proximity of a conformation to the experimental native structure and computed over the heavy backbone atoms). However, in cases where unbiased perturbation results in large *μ_|MM | _*values, changing *D *does effect how frequently near-native conformations are sampled (that is, the distribution of sampled minima). Figure 3 illustrates this for two representative systems by plotting, for different values of *D*, the distribution of *μ_|MM | _*values and the resulting distribution of lRMSD values. These results show that there is a distinct advantage to biasing the perturbation distance to *D *= 1Å or *D *= 2Å. Figures 3(a) and Figure 3(c) show that the frequency of small *μ_|MM | _*is larger when *D ∈ {*1, 2*}*Å vs. an unbiased perturbation. Figures 3(b) and Figure 3(d) show that the resulting ensembles contain more low-lRMSD conformations than the unbiased approach.

The effect of controlling *D *shown in Figure 3 is strongest on more heavily *β*-sheet proteins (those with native PDB ids 1dtdB, 1isuA, 1wapA, and 1hhp). On these proteins, an unbiased perturbation results in few small consecutive local minima distances. More near-native conformations are also obtained (though to a lesser extent) when *D *∈ *{*1, 2*} *for other proteins (with native PDB ids 1ail, 1sap, and 2h5nD). On these proteins, unbiased perturbation results in larger numbers of small consecutive local minima distances, but these proteins still benefit from enhanced sampling of neighboring local minima.

This enhanced sampling of near-native conformations can correspond to the BH search remaining in the same near-native region of the space; low *D *values could potentially cause the minimization to return to the previous minimum. In practice, this does occur for *D *= 1Å; however, when *D >*1Å, the search returns to previous local minima the same or less frequently than the unbiased approach.

#### MMC versus greedy search in minimization

Table 1 compares the greedy search (*T *= 0) to MMC searches with temperatures *T*_0_, *T*_1_, and *T*_2_. The lowest energies achieved under each setting are shown in columns 7-10. Results show that employing MMC as the minimization step achieves lower energy conformations than employing greedy search. In general, MMC with *T *= 0 achieves the lowest energy values for proteins less than 80 amino acids in length, while the lowest energies are achieved by the slightly higher temperature of *T*_1 _for longer proteins. This is possibly because in more complex rugged surfaces, small uphill moves allow reaching deeper minima.

**Figure 4:**
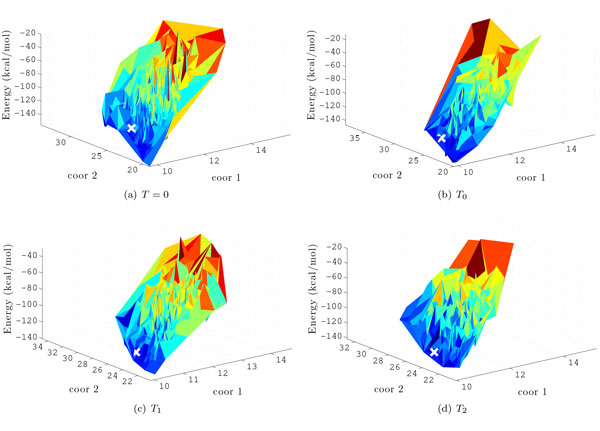
**The energy surface sampled for the protein with native PDB id **1fwp**is shown for each temperature *T ***. The × and y-axes represent projection coordinates based on interatomic distances within each conformation, and the z-axis represents the energy of each sampled local minimum. The white "x" indicates the location of the native structure in the energy surface.

The energy surface sampled by the BH framework for each given value of *T *is illustrated in Figure 4. The × and y-axes represent geometric projections of the conformations based on interatomic distances, and the z-axis represents the energy of each sampled local minimum. The Geometric projections are based on the mean interatomic distances between selected atoms (see [19] for more details). A large white "x" represents the location of the experimentally-determined native structure. Figure 4 illustrates that coarse-grained energy functions are noisy and result in surfaces that can deviate from the true protein energy surface. Columns 11-14 in Table 1 show, for each value of *T *, the lowest lRMSD to the native structure over Ω. The lowest lRMSD values obtained are comparable whether greedy or MMC search is employed in the minimization. This suggests that MMC minimization's ability to probe deeper into minima does not necessarily bring the BH search closer to the native structure.

The higher computational cost of each MMC minimization results in fewer sampled minima (total number of energy evaluations is fixed). Employing MMC in place of greedy search thus reduces the total number of hops in the BH trajectory by 50 to 70%, resulting in correspondingly fewer sampled minima. Columns 11-14 in Table 1, however, show that a lower number of sampled minima does not necessarily correlate with worse proximity to the native state. Focusing on a smaller ensemble of "interesting" local minima allows more computationally intensive refinement steps to focus resources more effectively.

### Multi-objective ensemble reduction

Multi-objective ensemble reduction proposed in the Methods section is evaluated by comparing its ability to retain near-native conformations to that of employing a threshold based on total energy. The use of the Pareto front and the Pareto count as metrics for ensemble reduction are evaluated in the "Pareto front reduction technique" and "Pareto count reduction technique" sections, respectively. To further evaluate the effectiveness of the multi-objective reduction technique, results are given for both the AMW energy function and the Rosetta coarse-grained energy function with "score3" weights. The ensembles Ω*_AMW _*and Ω*_Rosetta _*are generated for each target protein with the BH framework described in Methods employing unbiased perturbation and *T *= 0 for minimization.

The total energy for each conformation Ω is decomposed into individual energy terms described in Methods. Since multi-objective analysis is highly sensitive to the number of energy terms, the Rosetta energy terms are then combined into 5 groups so the number of terms is consistent between Ω*_AM W _*and Ω*_Rosetta _*in the multi-objective analysis. Grouping is done based on correlation between energy terms; more highly correlated terms are combined. In this work, the following energy term groupings are employed: {env, pair, cbeta, rg}, {vdw}, {cenpack}, {hs_pair}, {ss_pair, rsigma, sheet}. Since the terms ss_pair, rsigma, and sheet are primarily employed in the evaluation of beta sheets, their values often remain fixed for proteins without beta sheets or for proteins in which beta sheets are not accurately modeled. If one term remains fixed, then it is impossible for one conformation to dominate another using strong Pareto dominance as described in the Multi-objective ensemble reduction section. Therefore weak dominance is employed when performing multi-objective analysis on Ω*_Rosetta_*.

Tables 2 and 3 compare the ensemble reduced through a total energy threshold, Ω*_TE_*_(*n*)_, to the ensembles reduced by employing the Pareto front, Ω*_PF _*, and the Pareto count, Ω*_PC_*_(*n*)_, for the AMW and Rosetta energy functions. The ensemble Ω*_TE_*_(*n*) _is achieved by selecting a total energy threshold and removing all conformations with total energy greater than the threshold. The variable *n *is set to a particular percentage of Ω and a total energy threshold is chosen such that *| *Ω*_TE_*_(*n*)_*| *= n * | Ω *|*. Recall that the ensemble Ω*_PC_*_(*n*) _is constructed similarly to Ω*_TE_*_(*n*)_, however, the Pareto count is employed in place of total energy to rank conformations. For Ω*_PF _*only conformations in the non-dominated Pareto front are retained. For Ω*_PC_*_(*n*) _and Ω*_TE_*_(*n*)_, *n *can be set to any percentage of Ω, while the size of Ω*_PF _*is dictated by the size of the Pareto front for a given Ω.

**Table 2:** AMW multi-objective reduction technique.

AMW Energy Function									
	Native PDB Id	Ω*_PF _*reduction	Minimum lRMSD (Å)						
		(*r *= *|*Ω*_PF _|/|*Ω*|*)	Ω	Ω*_TE_*_(*r*)_	Ω*_PF_*	Ω*_TE_*_(5%)_	Ω*_TE_*_(10%)_	Ω*_PC_*_(5%)_	Ω*_PC_*_(10%)_
1	1dtdB	4%	7.2	7.9	7.7	7.9	7.7	7.7	7.7
2	1isuA	7%	6.0	6.2	6.5	6.4	6.2	6.2	6.2
3	1c8cA	4%	7.4	7.5	7.5	7.5	7.5	7.5	7.5
4	1sap	2%	6.5	7.6	7.5	7.4	7.2	7.4	7.2
5	1hz6A	2%	5.9	6.7	6.3	6.7	6.7	6.7	6.6
6	1wapA	2%	7.7	8.7	8.7	8.7	8.7	8.7	8.7
7	1fwp	7%	6.4	8.1	7.3	8.1	8.1	8.1	8.1
8	1ail	2%	3.4	6.8	5.9	5.8	4.2	4.7	4.4
9	1aoy	6%	5.7	6.9	6.6	6.9	6.5	6.8	6.5
10	1cc5	7%	5.6	8.6	7.0	8.7	8.6	8.6	8.1
11	2ezk	3%	4.4	8.0	7.3	7.7	7.1	7.2	7.1
12	1hhp	1%	10.7	12.0	12.0	11.6	11.6	11.6	10.8
13	2hg6	6%	8.6	10.8	10.5	11.6	10.8	10.9	10.8
14	3gwl	5%	4.2	4.7	5.2	4.7	4.7	4.7	4.7
15	2h5nD	7%	7.9	10.7	10.0	10.8	10.4	10.4	10.4

The minimum lRMSD to the native structure retained by each of the proposed multi-objective ensemble reduction techniques is given for the Ω generated with the AMW energy function. Column 3 gives the size of the Pareto front as a percentage of the size of Ω. Column 4 gives the minimum lRMSD to the native structure of any conformation in the Ω. Columns 5 and 6 give minimum lRMSD retained by Ω*_TE_*_(*r*) _and Ω*_PF _*, respectively, where *r *is the corresponding value from Column 3. Columns 7-10 compare the minimum lRMSD retained by Ω*_TE_*_(*n*) _and Ω*_PC_*_(*n*) _for thresholds of *n *= 5% and *n *= 10%.

**Table 3:** Rosetta multi-objective reduction technique.

Rosetta Energy Function									
	Native PDB Id	Ω*_PF _*reduction	Minimum lRMSD (Å)						
		(*r *= *| *Ω *_PF _|/| *Ω *|*)	Ω	Ω*_TE_*_(*r*)_	Ω*_PF_*	Ω*_TE_*_(5%)_	Ω*_TE_*_(10%)_	Ω*_PC_*_(5%)_	Ω*_PC_*_(10%)_
1	1dtdB	1%	6.7	10.8	9.1	10.6	10.2	10.2	8.6
2	1isuA	2%	6.5	8.9	8.6	8.9	8.6	8.0	7.5
3	1c8cA	2%	5.6	7.9	7.1	7.8	7.0	7.1	6.8
4	1sap	3%	6.1	7.4	7.1	7.4	6.8	6.8	6.6
5	1hz6A	3%	2.5	2.8	2.8	2.8	2.6	2.7	2.6
6	1wapA	1%	7.4	8.8	8.8	8.5	8.5	8.8	8.1
7	1fwp	3%	6.1	7.2	7.0	7.1	7.1	7.2	6.9
8	1ail	*>*1%	4.8	8.2	6.2	7.6	7.5	7.5	6.9
9	1aoy	2%	6.2	10.1	9.1	9.2	9.2	9.3	9.2
10	1cc5	1%	5.0	6.3	6.3	5.7	5.7	5.5	5.4
11	2ezk	1%	3.9	9.1	6.2	5.2	5.1	5.1	4.9
12	1hhp	3%	10.8	13.9	12.6	13.9	13.6	13.0	12.9
13	2hg6	2%	10.6	12.2	11.5	12.0	12.0	12.0	11.7
14	3gwl	1%	7.1	8.9	8.5	8.7	8.4	8.0	7.8
15	2h5nD	1%	8.9	13.0	10.4	12.3	12.1	12.2	11.4

The minimum lRMSD to the native structure retained by each of the proposed multi-objective ensemble reduction techniques is given for the Ω generated with the Rosetta energy function. Column 3 gives the size of the Pareto front as a percentage of the size of Ω. Column 4 gives the minimum lRMSD to the native structure of any conformation in the Ω. Columns 5 and 6 give minimum lRMSD retained by Ω*_TE_*_(*r*) _and Ω*_PF _*, respectively, where *r *is the corresponding value from Column 3. Columns 7-10 compare the minimum lRMSD retained by Ω*_TE_*_(*n*) _and Ω*_PC_*_(*n*) _for thresholds of *n *= 5% and *n *= 10%.

#### Pareto front reduction technique

Column 3 in Tables 2 and 3 shows that, when considering only conformations in the Pareto front, Ω*_PF _*, the size of Ω is reduced by over 90% across all target proteins and at least 95% for the majority of proteins. This shows that the Pareto front filter is a highly effective method for efficiently reducing the size of a large ensemble of decoy conformations. The difference between the average size of Ω*_PF _*employing AMW and Ω*_PF _*employing Rosetta is due to the the use of strong dominance for AMW and weak dominance Rosetta.

Columns 4-6 in Tables 2 and 3 show the minimum lRMSD to the native structure of all conformations in Ω, Ω*_TE_*_(*n*=*r*)_, and Ω*_PF _*, respectively. Here *r *is chosen such that *| *Ω*_TE_*_(*n*=*r*)_*| *= *| *Ω*_PF _|*, so a fair comparison can be made. While neither ensemble reduction technique is able to retain the lowest lRMSD to native conformations from Ω, comparison of columns 5 and 6 reveals that Ω*_PF _*retains conformations with lRMSDs to native not higher than Ω*_TE_*_(*r*) _for all but two proteins when employing the AMW energy function (Table and for all proteins when employing the Rosetta energy function (Table 3). This difference in lRMSD is significant (0.5Å or greater) for proteins with native PDB ids 1fwp, 1ail, 1cc5, 2ezk, 2h5nD for AMW and 1dtdB, 1c8cA, 1ail, 1aoy, 2ezk, 1hhp, 2hg6, 2h5nD for Rosetta.

**Figure 5:**
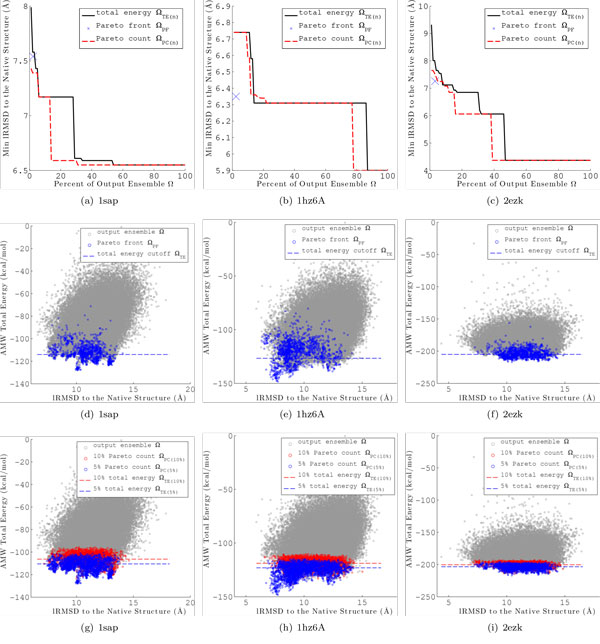
**Results for each of the proposed multi-objective ensemble filtering methods are shown for the AMW energy function on three representative proteins with native PDB ids 1sap, 1hz6A and 2ezk**. (a)-(c) show the minimum lRMSD to the native structure retained from the full ensemble Ω in the reduced ensembles Ω*_PC_*_(*n*) _(dashed red line) and Ω*_TE_*_(*n*) _(solid black line), for a given percentage *n *of the conformations in Ω. The minimum lRMSD retained by Ω*_PF _*is marked with a blue "X". (d)-(f) show the total energy versus lRMSD to the native structure for each conformation in the ensemble Ω. Conformations corresponding the the Pareto front, Ω*_PF _*, are colored in dark blue. The dashed line represents the energy cutoff such that *|*Ω*_TE_*_(*n*)_*| *= *|*Ω*_PF _|*. In (g)-(i), conformations are colored according to their Pareto count. Conformations in Ω*_PC_*_(*n*) _are colored in blue and red for *n *= 5% and *n *= 10%, respectively. The dashed lines represents the total energy cutoff for conformations in Ω*_TE_*_(*n*)_.

**Figure 6:**
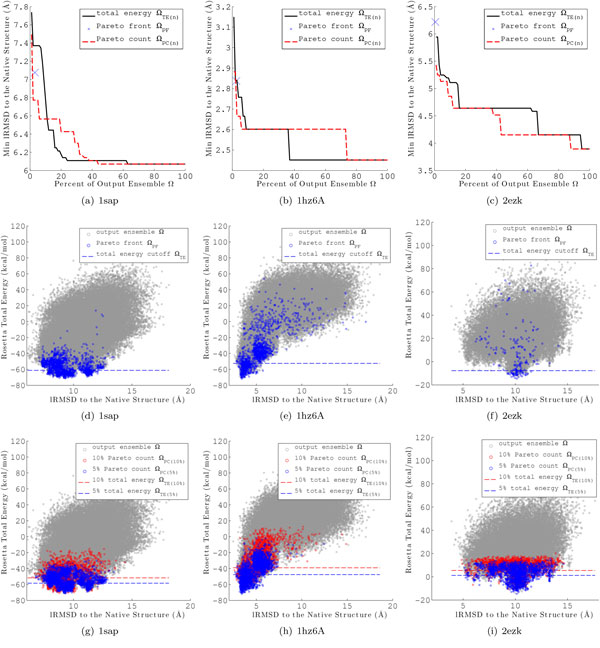
**Results for each of the proposed multi-objective ensemble filtering methods is shown for the Rosetta coarse-grained energy function on three representative proteins with native PDB ids 1sap, 1hz6A and 2ezk**. (a)-(c) show the minimum lRMSD to the experimentally determined native structure retained from the full ensemble Ω in the reduced ensembles Ω*_PC_*_(*n*) _(dashed red line) and Ω*_TE_*_(*n*) _(solid black line), for a given percentage *n *of the conformations in Ω. The minimum lRMSD retained by Ω*_PF _*is marked with a blue "X". (d)-(f) show the total energy versus lRMSD to the native structure for each conformation in the ensemble Ω. Conformations corresponding the the Pareto front, Ω*_PF _*, are colored in dark blue. The dashed line represents the energy cutoff such that *|*Ω*_TE_*_(*n*)_*| *= *|*Ω*_PF _|*. In (g)-(i), conformations are colored according to their Pareto count. Conformations in Ω*_PC_*_(*n*) _are colored in blue and red for *n *= 5% and *n *= 10%, respectively. The dashed lines represents the total energy cutoff for conformations in Ω*_TE_*_(*n*)_.

Merely looking at the minimum lRMSD to native structure retained does not tell the entire story. Figures 5(d) and 6(d) plot the energy versus lRMSD to native for each conformation in Ω for the AMW and Rosetta energy functions, respectively, for a representative protein with native PDB id 1sap. Conformations in Ω*_PF _*are highlighted in dark blue and a dashed line represents the energy cutoff for Ω*_TE_*_(*n*=*r*)_. For both energy functions, Ω*_PF _*retains lower lRMSD to native conformations than Ω*_TE_*_(*n*=*r*) _and Ω*_TE_*_(*n*=*r*) _loses significantly more of these near-native conformations. These results show that there is a clear advantage to employing the Pareto front over a total energy threshold to select conformations from Ω, and these results hold whether employing AMW or Rosetta.

Figure 6(e) represents an unusual case (illustrated by the protein with native PDB id 1hz6A) where the correlation between total energy and lRMSD to native is very high. High correlation is rarely the case for coarse-grained energy functions. We have specifically chosen to show 1hz6A here because Rosetta seems to capture well the true energy surface for this protein. For 1hz6A, a total energy threshold alone is sufficient for selecting decoy conformations with low lRMSDs, given this high correlation. In a blind prediction, the native structure is unknown and thus lRMSDs are not available. Thus, such cases are difficult to identify and the the Pareto front is still just as effective as a total energy threshold.

#### Pareto count reduction technique

Unlike Ω*_PF _*, the size of Ω*_PC_*_(*n*) _can be set for any desired value of *n*. Figures 5(a)-(c) (AMW energy function) and 6(a)-(c) (Rosetta energy function) show the minimum lRMSD to native for Ω*_PC_*_(*n*) _(dashed red line) and Ω*_TE_*_(*n*) _(solid black line) for *n *∈ {1, 2, 3*..*.100} on three selected proteins with PDB ids 1sap, 1hz6A, and 2ezk. The minimum lRMSD and size of Ω*_PF _*is also given for reference as a blue "X". Examination reveals that Ω*_PC_*_(*n*) _retains conformations with lRMSDs to the native structure as low or lower than Ω*_TE_*_(*n*) _for values of *n <*= 10% for all three proteins. This result is representative of all 15 target proteins investigated in this study. Columns 7-10 of Tables 2 and 3 give the minimum lRMSD for Ω*_TE_*_(*n*) _and Ω*_PC_*_(*n*) _for *n *= 5% and *n *= 10% for all 15 target proteins.

Figures 5(g)-(i) and Figure 6(g)-(i) plot the energy versus lRMSD to native for each conformation in Ω for the AMW and Rosetta energy functions, respectively, for same three representative proteins (PDB ids 1sap, 1hz6A, and 2ezk). Conformations in Ω*_PC_*_(5%) _and Ω*_PC_*_(10%) _are highlighted in blue and red, respectively. The dashed blue and red lines represent the total energy cutoffs for Ω*_TE_*_(5%) _and Ω*_TE_*_(10%)_, respectively. Examination of the common case of 1sap reveals that Ω*_PC_*_(*n*) _retains significantly more low-lRMSD conformations than Ω*_TE_*_(*n*) _for a given value of *n*. In the unusual case of 1hz6A, for which total energy is highly correlated with lRMSD, Ω*_PC_*_(*n*) _retains a similar range of low-lRMSD structures as Ω*_TE_*_(*n*) _does.

The protein with PDB id 2ezk represents a case where Ω*_PF _*is not effective at retaining low lRMSD structures. Figures 5(f) and Figure 6f show that the low-lRMSD conformations retained by Ω*_PF _*are outliers, particularly for the Rosetta energy function. Examination of Figures 5(i) and Figure 6(i) reveals that, for this difficult case, Ω*_PC_*_(*n*) _is still effective at sampling a range of low-lRMSD conformations. A similar results is seen for the protein with PDB id 1ail (data not shown here).

Taken together, these results show that employing multi-objective analysis to filter the output ensemble provides a distinct advantage over a total energy criterion. The ensemble size reduction is dramatic, yet non-outlier low-lRMSD conformations are still retained. In difficult cases the Pareto count metric retains low-lRMSD conformations even when the Pareto front does not.

## Conclusions

This work shows that careful realizations of the BH framework can provide both rapid sampling and enhanced sampling of the protein conformational space. In addition to previous work, where a simple realization of the BH framework was shown competitive in terms of obtaining lowest lRMSDs to the native structure comparable to state-of-the-art MC-based methods [25], this work shows the high sampling capability and the diversity of the decoy ensemble obtained by BH-based algorithms. We draw attention to the ability of the algorithm to obtain many non-native conformations of low energies, which is a hallmark of algorithms with high sampling capability [47,48].

This work provides a deeper understanding of the BH framework and its premise for obtaining an effective decoy ensemble. The two algorithmic components of the framework, perturbation and minimization, are analyzed in detail, and effective implementations are offered to control the exploration for the purpose of obtaining a diverse decoy ensemble. Results show that the distance between consecutively-sampled local minima is directly affected by the perturbation distance. Our experiments demonstrate that by biasing perturbation distance, one can enhance sampling of near-native decoys in the BH framework. Moreover, a simple greedy search was shown just as effective at sampling near-native conformations as a more computationally intensive MMC trajectory.

Employing short greedy searches for minimization is appealing, as it allows sampling a significantly larger number of local minima than longer MMC trajectories. This larger ensemble provides a broad view of low-energy local minima in the coarse-grained energy surface, but inaccuracies in the energy function do not allow relating near-native conformations with the lowest-energy minima. To deal with this issue, we present an ensemble reduction technique based on multi-objective analysis. Metrics based on the Pareto front and Pareto count are proposed, and analysis is performed on the decoy ensemble generated by our BH framework employing either the AMW or the Rosetta coarse-grained energy functions.

For all of proteins investigated in this work, the Pareto-based reduction technique is highly effective at reducing the ensemble while still maintaining non-outlier near-native conformations. Multi-objective metrics based on Pareto dominance are an ideal choice because they can be computed online and have lower computational complexity than structure-based clustering algorithms. Future work will investigate this setting to further enhance sampling capability while retaining an informative conformational ensemble.

## Competing interests

The authors declare that they have no competing interests.

## Authors' contributions

BSO suggested the methods and the performance study in this manuscript and drafted the manuscript. AS guided the study, provided comments and suggestions on the methods and performance evaluation, and improved the manuscript writing.

## Declarations

The publication costs for this article were funded by the corresponding author AS through NSF Grant Nos. 1016995 and 1144106.

This article has been published as part of *Proteome Science *Volume 11 Supplement 1, 2013: Selected articles from the IEEE International Conference on Bioinformatics and Biomedicine 2012: Proteome Science. The full contents of the supplement are available online at http://www.proteomesci.com/supplements/11/S1.
